# Bilateral rectus femoris intramuscular haematoma following simultaneous quadriceps strain in an athlete: a case report

**DOI:** 10.1186/1752-1947-4-56

**Published:** 2010-02-18

**Authors:** Konstantinos Natsis, Christos Lyrtzis, Georgios Noussios, Efthymia Papathanasiou, Nikolaos Anastasopoulos, Trifon Totlis

**Affiliations:** 1 Department of Anatomy, Medical School, Aristotle University of Thessaloniki, Greece

## Abstract

**Introduction:**

Bilateral rectus femoris haematoma following a simultaneous strain of the quadriceps muscles is a very rare condition.

**Case presentation:**

We report the case of a 21-year-old Greek Caucasian female rowing athlete who was injured on both thighs. She complained of pain and inability to walk. Physical examination revealed tenderness over the thighs and restriction of knee movement. The result of a roentgenogram was normal, and there was no evidence of fracture or patella displacement. Magnetic resonance imaging revealed haematoma formation in both the rectus femoris muscles. The diameters of the left and right haematomas within the muscles were 6 cm and 5 cm, respectively. Therapeutic approaches included compression bandages, ice application, rest, elevation, and administration of muscle relaxant drugs. Active stretching and isometric exercises were performed after three days. The patient was able to walk using crutches two days after the initiation of treatment. On the seventh day, she had regained her full ability to walk without crutches. Non-steroidal anti-inflammatory drugs were administered on the fifth day and continued for one week. Six weeks later, she had pain-free function and the result of magnetic resonance imaging was normal. She was able to resume her training programme and two weeks later, she returned to her previous sport activities and competitions.

**Conclusion:**

There are references in the literature regarding the occurrence of unilateral quadriceps haematomas following strain and bilateral quadriceps tendon rupture in athletes. Simultaneous bilateral rectus femoris haematomas after a muscle strain is a rare condition. It must be diagnosed early. The three phases of treatment are rest, knee mobilization, and restoration of quadriceps function.

## Introduction

Traumatic musculoskeletal pathology is frequent in athletes. Muscle strains are the most common injuries, especially in sports involving running. They are defined as an indirect injury to a muscle that produces tension overload in a passive muscle or eccentric overload in an actively contracting muscle [1]. They vary from mild or first degree to muscle tear or third degree [2]. Severe muscle strains can lead to haematoma formation. The most frequent cause of partial or complete muscle rupture is its eccentric overload [3,4]. On the other side, the contusions result from a direct impact against the muscle or from muscle overstressing [2]. These lesions usually heal spontaneously and leave no sequel, but they may take several months to heal as well.

The classification of strains is based on their severity. A mild (first degree) strain describes a rupture of a few fibres with minor loss of strength or restriction of movement. Active movement or passive stretching produces a mild aching discomfort. Meanwhile, a moderate (second degree) strain involves greater damage of muscle. The pain is aggravated by any attempt to move the muscle and there is clear loss of strength. Lastly, a severe (third degree) strain involves a complete disruption of the muscle, thus resulting in total lack of muscle function [5]. The team physician must be able to predict how long the healing process will take in order to avoid a long period of inactivity. At the same time, he must be able to protect the patient from a recurrent tear. Unilateral quadriceps haematoma following strain in athletes and bilateral quadriceps tendon rupture at once have been reported in the literature [6]. We report a case of simultaneous bilateral rectus femoris haematoma following quadriceps strain in an athlete.

## Case presentation

A 21-year-old Greek Caucasian female rowing athlete was injured on both thighs during field training. She had to train in sprint as part of her field training program. Upon acceleration, she experienced severe pain on both thighs and fell down. She continued to suffer from severe pain on the anterior surface of her thighs and tenderness with any attempt of movement. She was also unable to stand up and walk. Her trainer observed swelling and loss of function immediately after the trauma and he tried to control the pain with compression dressing and ice packs while they were in the field. She was later brought to our clinic by an ambulance.

On physical examination, an oedema was found on the anterior surface of her thighs. The pain was continuous and aggravated on palpation of the quadriceps muscle and any knee movement. There was no gap in quadriceps continuity. Her active and passive knee flexion and extension were restricted and painful. She was not able to perform an isometric quadriceps contraction with her knee in full extension. The active knee's range of movement was 40° for the right and 55° for the left. The passive range of movement was the same because of the pain. We checked the pulse of her periphery arteries with a Doppler ultrasound machine and we found it normal. After the physical examination a roentgenogram was performed. The roentgenogram result was negative for fracture and the patella was not displaced.

**Figure 1:**
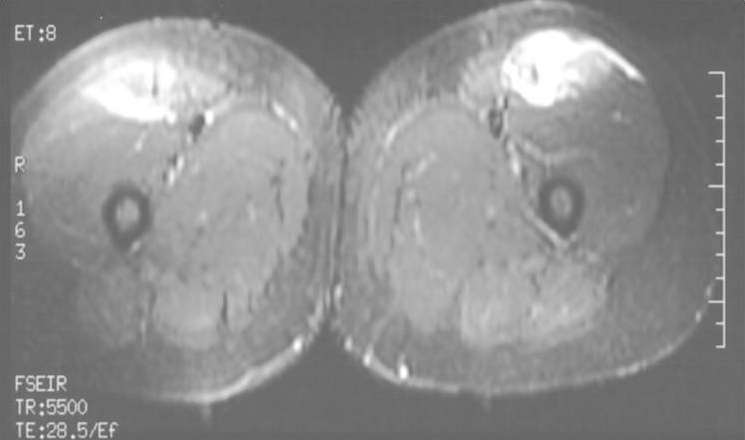
**Horizontal MRI section imaging the haematoma on both thighs**.

**Figure 2:**
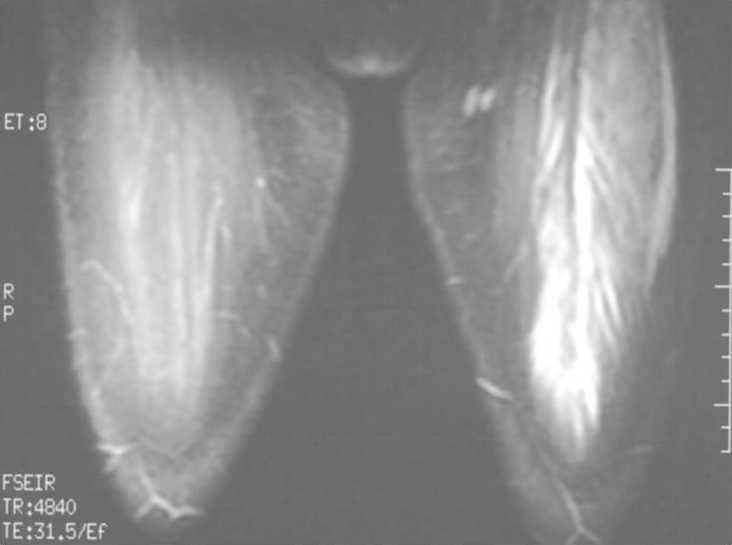
**Coronal MRI section imaging haematoma of the left thigh**.

**Figure 3:**
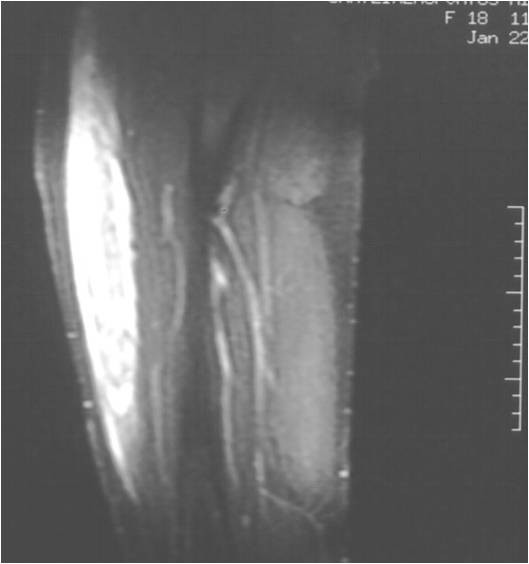
**Coronal MRI section imaging haematoma of the right thigh**.

Ultrasonography revealed haematoma formation on both her rectus femoris muscles, and magnetic resonance imaging (MRI) was then performed to estimate the size of the haematomas and to evaluate the surrounding soft tissues (Figure 1, Figure 2, Figure 3). The diameters of the left and right haematomas within the muscles were 6 cm and 5 cm, respectively.

Based on physical and MRI examinations the strains were classified as second grade or moderate. We examined the athlete to exclude the occurrence of compartment syndrome and we checked her coagulation profile by blood laboratory examination. We did not find any bleeding diathesis. She did not report any connective tissue disorder in her family and any use of anabolic steroids. Our patient was treated conservatively.

The treatment included compression bandage, ice application, and rest and elevation for the first 48 hours. Muscle relaxant drugs were administered for 1 week in maximum doses. We administered non-steroidal anti-inflamatory drugs (NSAID) on the 5^th ^day to reduce the pain and to avoid the development of myositis ossificans. Afterwards, we applied isometric exercises and active stretching of the muscle within our patient's pain limits. She was instructed to perform active, pain-free quadriceps stretching 15 times a day and pain-free isometric quadriceps strengthening exercises. Two days later she started to walk using crutches.

On the 7th day our patient started stretching exercises, and she was able to walk without crutches. The active and the passive ranges of movement of her knees were bilaterally the same. The active range of movement was 110° and the passive was 120°. The three phases of treatment were rest, knee mobilization, and restoration of quadriceps function. The goals included pain-free knee flexion and extension and rapid, unrestricted return to her full athletic activities.

**Figure 4:**
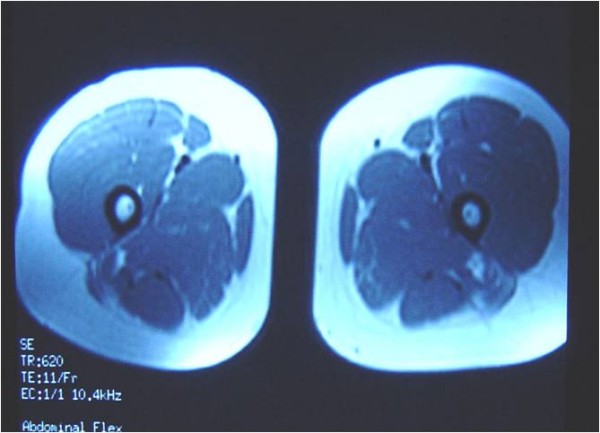
**Horizontal MRI section six weeks later**.

**Figure 5:**
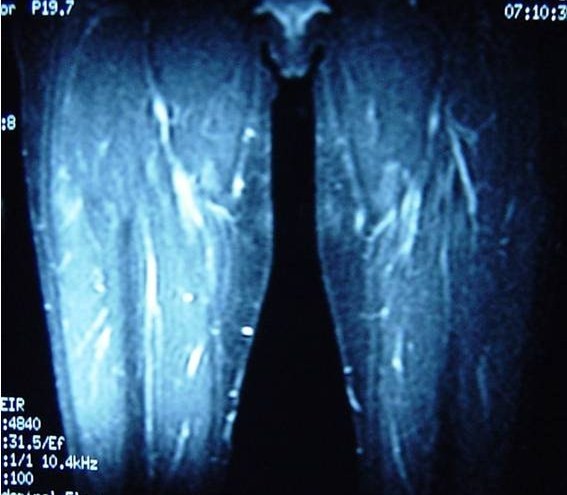
**Coronal MRI section six weeks later**.

Six weeks later MRI result was normal and she had regained a full pain-free range of movement (Figure 4, Figure 5). She started training and two weeks later returned to her old sport activities and competitions. No recurrence of symptoms was observed during the follow-up examination. A follow-up radiographic examination was performed on the third and sixth month after the injury to exclude the development of myositis ossificans.

## Discussion

Quadriceps strains frequently occur in athletes while training or participating on a race. The rectus femoris at the myotendinous junction is the most susceptible to injury because of its superficial location, biarticular course, most oftenly eccentric action, and higher content of type II fibres [1,3]. Other muscles with these characteristics are the hamstrings and the gastrocnemius muscles [1]. The formation of haematomas following muscle strain cannot be prevented. Fatigue, inflexibility, poor coordination and intrinsic tightness are factors that contribute to muscle overload [1,2]. Acute rectus femoris strains are usually located distal to the thigh, in contrast to chronic injuries that are more common near the muscle origin [7].

Medical imaging can define the precise location and severity of muscle traumas and detect critical elements that will delay complete repair. Ultrasonography is an efficacious and inexpensive imaging technique for analyzing muscular trauma [8]. It provides sufficient examination of muscle fibres, tendons and aponeurosis, but the visualization of deep structures is limited. MRI is the imaging technique of choice for the evaluation of acute musculotendinous injuries [3] as it makes the appearance of haematomas variable depending on the age of the haematoma [9]. It is a useful examination method for diagnosing soft-tissue injuries in cases where swelling or other soft-tissue abnormalities obscure the examination or preclude the use of more routine diagnostic modalities. In addition, MRI is most sensitive in evaluating the healing process and should thus be performed before the patients return to their exercise routine [10].

Haematoma formation in the quadriceps muscle rarely leads to increased pressure (41 mmHg to 80 mmHg) within the muscle compartment, and thus to the development of compartment syndrome [11]. Compartment syndrome comprises severe pain a few hours after the trauma, which deteriorates during passive movement and hypesthesia or paresthesia distal to the thigh [2]. The only indication for fasciotomy and haematoma evacuation is the development of compartment syndrome [12]. Any surgical intervention in the acute phase of haematoma is contraindicated [13].

Quadriceps haematoma predisposes to the development of myositis ossificans. Myositis ossificans occurs after a strain in deep muscles. In traumatic myositis ossificans, the bone is deposited within a muscle as a result of haematoma [2]. King identifies different mechanisms for the formation of new bones within the injured muscle [14]. The hospitalization and disability time is longer in patients with myositis ossificans [13]. The treatment of muscle strains consists of the rest, ice application, compression and elevation (RICE) protocol [15]. In an experimental study by Walton *et al*., it was demonstrated that changes in tissue temperature are depth dependent after the application of ice packs [16]. Passive stretching and massage should be avoided until the patient restores a painless range of motion [1,7].

There are many treatment protocols and the most known is the one reported by Jackson and Feagin [17]. Other authors propose modifications of this protocol, such as resting of the injured leg in flexion versus extension and early flexion exercises versus extension [15]. According to other studies, placing and holding the knee in 120° of flexion immediately following a quadriceps strain helps to shorten the time of return to unrestricted full athletic activities [18]. However, there is not a widely acceptable protocol and further evidence-based research is needed especially when it comes to rehabilitation programmes [19].

Older athletes require prolonged missed playing time [20]. The high risk of recurrence of soft tissue injuries in athletes is attributed to their early return in training and sport activities before the injury has completely healed [20]. The athlete should not be allowed to return to sport activities until he can demonstrate muscle flexibility and strength [2].

## Conclusion

Quadriceps strain often occurs in athletes. It usually develops in the quadriceps muscles, and the rectus femoris is the most susceptible. Unilateral quadriceps haematomas following strain in athletes and bilateral quadriceps tendon rupture have been reported in the literature. The team physician must be informed about the possibility of simultaneously bilateral rectus femoris hematoma after a muscle strain in order to stress the importance of diagnosing this condition early. The three phases of its treatment are rest, knee mobilization, and restoration of quadriceps function.

## Consent

Written informed consent was obtained from the patient for publication of this case report and any accompanying images. A copy of the written consent is available for review by the Editor-in-Chief of this journal.

## Competing interests

The authors declare that they have no competing interests.

## Authors' contributions

KN performed the patient's treatment and gave the final approval for submitting the manuscript. CL participated in designing the study and conceived and drafted the manuscript. GN participated in the literature research. EP participated in the study design and literature research. NA participated in the literature research. TT participated in the literature research. All authors read and approved the final manuscript.
